# Molecular Population Genetics of Aspen Mosaic-Associated Virus in Finland and Sweden

**DOI:** 10.3390/v15081678

**Published:** 2023-08-01

**Authors:** Shaheen Nourinejhad Zarghani, Rim Al Kubrusli, Serghei Iancev, Risto Jalkanen, Carmen Büttner, Susanne von Bargen

**Affiliations:** 1 Division Phytomedicine, Faculty of Life Sciences, Albrecht Daniel Thaer-Institute of Agricultural and Horticultural Sciences, Humboldt-Universität zu Berlin, Lentzeallee 55-57, 14197 Berlin, Germany; nourines@hu-berlin.de (S.N.Z.); kubruslr@hu-berlin.de (R.A.K.); carmen.buettner@agrar.hu-berlin.de (C.B.); 2 Silva Lapponica, 96460 Rovaniemi, Finland

**Keywords:** emaravirus, –ssRNA, full length, PCR–RFLP, phylogeny, diversity, RT–PCR, cloning, forest, ornamental trees

## Abstract

Aspen mosaic-associated virus (AsMaV) is a newly identified *Emaravirus*, in the family *Fimoviridae*, *Bunyavirales*, associated with mosaic symptoms in aspen trees (*Populus tremula*). Aspen trees are widely distributed in Europe and understanding the population structure of AsMaV may aid in the development of better management strategies. The virus genome consists of five negative-sense single-stranded RNA (–ssRNA) molecules. To investigate the genetic diversity and population parameters of AsMaV, different regions of the genome were amplified and analyzed and full-length sequence of the divergent isolates were cloned and sequenced. The results show that RNA3 or nucleoprotein is a good representative for studying genetic diversity in AsMaV. Developed RT–PCR–RFLP was able to identify areas with a higher number of haplotypes and could be applied for screening the large number of samples. In general, AsMaV has a conserved genome and based on the phylogenetic studies, geographical structuring was observed in AsMaV isolates from Sweden and Finland, which could be attributed to founder effects. The genome of AsMaV is under purifying selection but not distributed uniformly on genomic RNAs. Distant AsMaV isolates displayed amino acid sequence variations compared to other isolates, and bioinformatic analysis predicted potential post-translational modification sites in some viral proteins.

## 1. Introduction

Aspen trees (*Populus tremula*) are widely distributed across Europe and play an important ecological and economic role in the region. These trees are particularly abundant in Northern Europe, including Finland, and Sweden. Aspens are known for their adaptability, fast growth, and ability to colonize disturbed areas [1]. They also provide a wide range of ecosystem services, including habitats for wildlife, carbon sequestration, soil stabilization, and water regulation [2,3,4]. In addition to their ecological significance, aspen trees have cultural and historical importance to people worldwide. In addition to fibers being used in the production of pulp as strand board, solid wood, and furniture, aspen wood is used in many specialty products, for instance, pencils, skis, sauna benches, and coffins [2].

Recently, it has been reported that aspen mosaic-associated virus (AsMaV) is associated with leaf symptoms, such as mottle, yellow blotching, variegation, and chloroses along veins [5]. Our surveys of many years have led to the conclusion that the virus is probably associated with the decline in aspen trees [5]. AsMaV is a member of the virus genus *Emaravirus*, which includes other plant pathogens such as the European mountain ash ringspot-associated virus and the rose rosette virus [6]. AsMaV has a segmented negative-sense single-stranded RNA (–ssRNA) genome designated as RNA1–RNA5. The genome of AsMaV consists of five –ssRNA molecules. The RNA1 (7.1 kb) encodes for the viral RNA-dependent RNA polymerase (RdRP, 268.2 kDa) and it is involved in virus replication. The RNA2 (2.3 kb), RNA3 (1.6 kb), RNA4 (1.6 kb), and RNA5 (1.3 kb) encode for the glycoprotein precursor (GPP, 73.5 kDa), viral nucleocapsid protein (NP, 35.6 kDa), a putative movement protein (MP, 41.0 kDa), and a protein of unknown function (P28, 28.1 kDa), respectively [5].

RNA viruses, which account for the majority of plant viruses, have high mutation rates due to the error prone nature of viral replicase or RdRP followed by other mechanisms such as recombination and reassortment [7,8,9,10,11]. The introduced mutations during the replication cycle of a virus are mostly deleterious for the virus, but sometimes they may lead to the generation of new isolates with altered phenotypes, such as host range, symptom severity, and transmission efficiency [12,13,14,15,16,17]. The effect of these phenomena in shaping of plant viruses has been reviewed [18,19,20,21,22,23,24,25,26,27].

The genetic diversity of plant viruses is important for understanding their evolution, spread, and potential of emerging new isolates may that are resistant to existing control measures. The genome of the emaraviruses consist of at least four –ssRNA (RNA1–4, core genome) but could extend to twelve segments [6,28]. The genetic diversity of a few members of emaraviruses, including fig mosaic virus (FMV) [29,30], blackberry leaf mottle-associated virus (BLMaV) [31], maple mottle-associated virus (MaMaV) [32], raspberry leaf blotch virus (RLBV) [33,34], rose rosette virus (RRV) [35], jujube yellow mottle-associated virus (JYMaV) [36], Perilla mosaic virus (PerMV) [37], European mountain ash ringspot-associated virus (EMARaV) [38], and High Plains wheat mosaic virus (HPWMoV) [39], has been studied. These studies have utilized one or more partial or complete viral genomic RNA(s), among which RNA3 has been identified as the main genomic region for studying the genetic diversity of emaraviruses followed by RNA4 and partial RNA1, all belonging to the core genome of emaraviruses. It should be mentioned that NP has been used not only in the genetic diversity of emaraviruses but also in all –ssRNA viruses [40,41,42,43]. Moreover, the NP is important in immunological tests for the identification of –ssRNA viruses and has been used in human [44] and plant viruses such as emaraviruses [45]. The aim of this study is the evaluation of the AsMaV diversity of populations originating from Finland and Sweden where the virus is widely distributed [5], based mainly on RNA3, RNA4, and partial RNA1 which encode for NP, MP, and RdRp, respectively.

## 2. Materials and Methods

### 2.1. Plant Source

A total number of 83 leaf samples of aspen trees showing mosaic, mottling, vein yellowing, line patterns, and chlorotic ring spots were collected in the summer of 2019. The samples were gathered from various forest sites in Finland, namely Oikarainen-Rovaniemi, Santa Claus Holiday Village, Hyypiöoja-Rovaniemi, Lustila-Rovaniemi, Kivitaipale-Rovaniemi, Petäjäinen-Rovaniemi, Mattinen-Tervola, Maula-Keminmaa, Peura, Tervalo, Lautiosaari-Keminmaa, Ritikka-Kemi, Kuninkaansaari-Helsinki, Hyypiökivalo, and Vallisaari-Helsinki. Additionally, sampling was conducted in several forest sites in Sweden, including Skede-Kråkshult, Ångelsjön Nature Reserve, Fagertärn-road, Sunnersberg-Fiskbäck, Kiså, Stora-Anrås, and Boliden. These samples were immediately transferred to the lab and kept at −70 °C for later use. The complete list of collected samples, along with their corresponding symptoms and geographical origin, is provided in Supplementary Table S1.

### 2.2. Primers, Detection of AsMaV by RT–PCR

A minimum of five symptomatic leaves from the same tree were selected and neatly piled up. Then, approximately 1–2 cm^2^ of the leaves (0.2–0.3 g) were used for RNA extraction. Total RNA was isolated according to the protocol described by Boom et al. [46] and 1.5 µg of the extracted RNA was applied as a template for cDNAs synthesis using random hexamers and Maxima H Minus Reverse Transcriptase kit (Thermo Scientific™, Waltham, MA, USA) following the protocol provided by the manufacturer. The presence of AsMaV in the collected samples was confirmed through a series of RT–PCRs employing specific primers (Supplementary Table S2). These primers were designed to hybridize with AsMaV-RNA3 to RNA5, following established method described elsewhere [5,47]. To design the novel primers, we utilized the RNA1 to RNA5 sequences of AsMaV isolates E55089 (GenBank accession No. LR74246, LR742462–65) as a reference. The detailed characteristics of these newly designed primers are provided in Supplementary Table S2. Notably, the isolate E55089 and its corresponding genomic molecules were used as a standard for nucleotide and amino acid positioning.

### 2.3. AsMaV–RNA3 RT–PCR–RFLP

The primer PDAP–213 [48], which hybridizes to the conserved 13 terminal nucleotides of all emaraviruses, was used to amplify RNA3 and RNA4 in at least 9 isolates. These PCR products were cloned and sequenced and the resulting data were used for designing specific primers for amplification of almost full-length RNA3 and RNA4 (Supplementary Table S2). Therefore, primer pair AsMaV–R3–18–s/AsMaV–R3–end–as was used for the amplification of almost full length RNA3 in remaining AsMaV isolates. The RT–PCR products were gel-extracted and subjected to restriction fragment length polymorphism (RFLP) using the FastDigest *AanI* restriction enzyme (Thermo Scientific™, Waltham, MA, USA). Isolates were grouped based on the RFLP profiles (restrictotypes) and sampling location. At least three samples from each location and each restrictotype were selected for downstream analysis.

### 2.4. Amplification of Different Genomic Regions

The primer pairs AsMaV–R4–12–s/AsMaV–R4–end–as and AsMaV–R1AE–1–s/AsMaV–R1A–1627–as were used for amplification of almost full-length RNA4 and partial RNA1 (nucleotides 1–1637), respectively. The sequences of primers are presented in the Supplementary Table S2. The primer PDAP–213 [48], which is hybridizing to the conserved 13 terminal nucleotides of all emaraviruses, was used to amplify RNA2, RNA3, RNA4, and RNA5 of isolates E58627 and E58632 according to the method described by [48]. RNA1 of isolates E58627 and E58632 was amplified in overlapping fragments using the primer pairs of AsMaV–R1AE–1–s/AsMaV–R1A–1627–as, AsMaV–R1B–1541–s/AsMaV–R1B–3107–as, AsMaV–R1C–2953–s/AsMAV–R1C–4492–as, AsMaV–R1D–4435–s/AsMaV–R1D–6011–as, and AsMaV–R1E–5968–s/AsMaV–R1AE–1–s. All PCRs were set up using the VELOCITY DNA Polymerase, a proof-reading DNA polymerase (BioCat GmbH, Heidelberg, Germany) in a 50 µL volume according to the manufacturer’s recommendations. RT–PCR products were separated by agarose gel electrophoresis, and expected DNA fragments were cut out and isolated using the GeneJET Gel Extraction Kit (Thermo Scientific, Waltham, MA, USA). The isolated fragments were subjected to direct sequencing or Sanger sequencing after cloning.

### 2.5. Cloning, Sequencing, and Data Analysis

CloneJET PCR Cloning Kit (Thermo Scientific) was used for cloning RT–PCR products following the manufacturer’s protocol. The sequencing was performed at Macrogen Europe (Amsterdam, The Netherlands). Sequence alignments were performed by MUSCLE program implemented into Geneious Prime ver. 2022.2.2 (12 December 2021) (https://www.geneious.com/). The best-fitting model of nucleotide substitution for each alignment was determined using Find best DNA/Protein models implemented in MEGA11 software (12 December 2021) [49]. The neighbor-joining (NJ, as a distance-based method) trees were generated using MEGA11 [49] and consensus maximum parsimony (CMP, as an evolutionary-based method) trees constructed by Phylogeny Inference Package (Phylip) version 3.65 software [50]. A bootstrap value of 1000 replicates for each node of the generated trees was calculated, and the trees were viewed using TreeDyn (available online at http://www.treedyn.org, 12 December 2021). The branches with bootstrap values lower than 70% were collapsed. Population genetic parameters were estimated by DnaSP version 6.12.03 software [51]. The corresponding regions of fig mosaic virus (FMV), pistacia virus B (PiVB), and pigeonpea sterility mosaic virus *2* (PPSMV–2) were used as outgroups in the analyses. RNAfold web server (http://rna.tbi.univie.ac.at//cgi-bin/RNAWebSuite/RNAfold.cgi, 12 April 2023 ) was used for prediction of the secondary structure of RNA based on the minimum free energy (MFE) and Centroid secondary structure with the minimum base pair distance prediction. RDP5 [52] was used for the recombination analysis. SignalP–6.0 (https://services.healthtech.dtu.dk/services/SignalP-6.0/, 12 April 2023), Prediction of Signal Peptides and their cleavage sites in all domains of life, was used for the prediction of cleavage sites of AsMaV GPP and NetNGlyc–1.0 (https://services.healthtech.dtu.dk/services/NetNGlyc-1.0/, 12 April 2023) was used for identification of post-translational modifications of the GPP. PopART software was used for generating the distribution of the restrictotype distribution of a geographical map [53,54,55].

## 3. Results and Discussion

### 3.1. Detection of AsMaV and RT–PCR–RFLP

RT–PCR was performed using detection primers for AsMaV [5] on total RNA extracted from collected plant material, resulting in the amplification of expected products of approximately 317, 288, and 672 bp for AsMaV RNA3–5, respectively, in 34 samples from Sweden and 49 samples from Finland. Using the primer set, AsMaV–R3–18–s/AsMaV–R3–end–as and AanI restriction enzyme, electrophoresis of digested RT–PCR products revealed seven different restrictotypes (Table 1). Restrictotype VII was the most prevalent and dominant type found in all tested areas, except in Mattinen-Tervola (Finland) and Skede-Kråkshult (Sweden), where only Type IV and Type VI profiles were found, respectively. Notably, Kuninkaansaari-Helsinki and Vallisaari-Helsinki (Finland), the two small islands connected by a narrow bridge, showed five different restrictotypes (Type II, IV, VI, and VII). Similarly, Kiså (Sweden) showed three different restrictotypes (Type III, V, and VII). Identical restrictotypes were identified in both countries, suggesting the fixation of these mutations in RNA3. At least three samples from each location and each of restrictotypes were selected for sequencing and further analysis (in total 27 isolates). There was no correlation between geographical origin and restrictotype, and the distribution of restrictotypes was not uniform across all studied geographical regions.

### 3.2. Genetic Diversity and Phylogeny of AsMaV Isolates Based on RNA3

**Figure 1:**
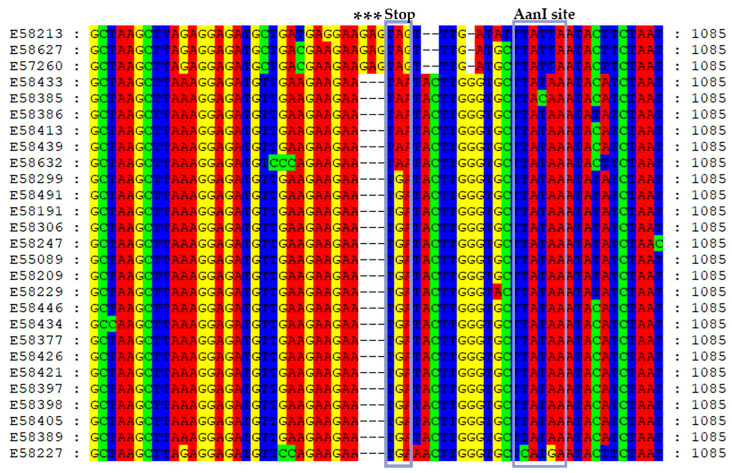
Nucleotide sequence alignment of AsMaV ORF3 termini reveals the presence of three distinct stop codons in the sequenced isolates. To highlight these stop codons, they have been marked with gray rectangular regions accompanied by the label “Stop” at the top. Notably, an indel event involving the insertion of three nucleotides, specifically “GAG”, has been observed in isolates E58213, E58627, and E57260, occurring immediately prior to the termination codon. These significant positions have been duly identified using asterisks. Furthermore, experimental confirmation of the AanI restriction site downstream of the respective stop codons has been obtained, and their corresponding positions in the alignment are indicated as AanI site.

The length of AsMaV–RNA3 in sequenced isolates was 1588–1589 nucleotides in size, with 86.8–100% sequence identity among the isolates. Isolates E58213 (Sweden), E58627, and E57260 (Finland) were the most divergent isolates. The 3′UTR region exhibited the highest level of sequence diversity (Table 2). The length of AsMaV RNA3 was previously reported to be 1587 nucleotides (nts) for isolate E55089 (GenBank Accession number LR742463). In 80% of the sequenced isolates, RNA3 was 1588 nts long, while in the remaining isolates, it was 1589 nucleotides in size. The ORF3 started at nt 101 and ended at nt 1057 with the exception of isolates E58627, E58213, and E57260 which ended at nt 1060 (Figure 1). Consequently, the length of the P3 gene or NP gene was 957 or 960 nucleotides encoding for 318–319 amino acids (aa), respectively. As previously published, RNA3 has a very long 3′ UTR, i.e., 530 nts [5]. Three different stop codons were detected in AsMaV ORF3, with the amber stop codon (UGA) being the most prevalent in the majority of isolates (18 out of 27). The ochre (UAA) and opal (UGA) stop codons were detected in six and three isolates, respectively (Figure 1). Stop codons have varying termination efficiencies (UAA > UAG > UGA). In plant viruses, either amber (UAG) or opal (UGA) stop codons are read through; there are no examples of read-through of the natural ochre (UAA) stop codon [56]. The presence of several stop codons in different frame shifts downstream of the ORF3 might be denoting the importance of the NP size.

The three conserved motifs previously reported for the NP of emaraviruses (NXXSXNXXXA, NXLA, and GYEF) [57,58] were also found in AsMaV–NP. The first motif identified in AsMaV–NP was NIVSFNKACA_131–140_ which was present in all sequenced isolates, except for E58446 and E58434 where the last amino acid was P instead of A. The motif NRLA and GYEF were found in positions 179–182 and 200–203, respectively. The first motif has not been reported for (PerMV) and motif GYEF was replaced with GVEF_159–162_. The aa X_139_ was also R in the motif NXLA_138–141_ of PerMV. In the AsMaV and PerMV, there is a 21 aa distance between the second and third motif, which appears to be conserved among the emaraviruses and essential for the folding or functioning of the protein. However, the distance between the first and second motif (35–39 amino acids) was not conserved among the emaraviruses.

Sequence data confirmed the presence of AanI site in RNA3 at the positions 627–632, 1069–1074, 1142–1147, 1406–1411, and 1473–1478 based on the nt sequence of the isolate E55089 (GenBank Acc. No. LR742463). Among these restriction sites, only one of them was placed in the ORF3 (nts 627–632), and the rest of the AanI restriction sites were detected in the 3′ UTR. The first nt of AanI restriction site (T_628_) was placed in the position of nt 627–632 (5′–CTTATAATC–3′) and is the second codon position of aa leucine. Therefore, the second nucleotide position of the AanI site is the third-codon position of leucine in which any changes in that position do not result in non-synonymous substitutions. Interestingly, the nt 627–632 AanI site is adjacent to the emaravirus motif NxLA (Table 1). The rest of the AanI sites are spanned in 3′ UTR which was the largest untranslated region of AsMaV and the most divergent part of RNA3. Searching for the secondary structure of RNA in the long 3′ UTR based on the minimum free energy (MFE) and Centroid secondary structure with the minimum base pair distance prediction (http://rna.tbi.univie.ac.at//cgi-bin/RNAWebSuite/RNAfold.cgi, 12 April 2023) in this region showed the potential complementary regions forming stem-loop structures. Interestingly, nt 1272–1358 in the 3′ UTR was the most conserved AT-rich region. This region did not harbor any secondary structure which might explain the toleration of mutations resulting in variations in this region. A similar sequence was not found when we applied BLAST search. 

**Figure 2:**
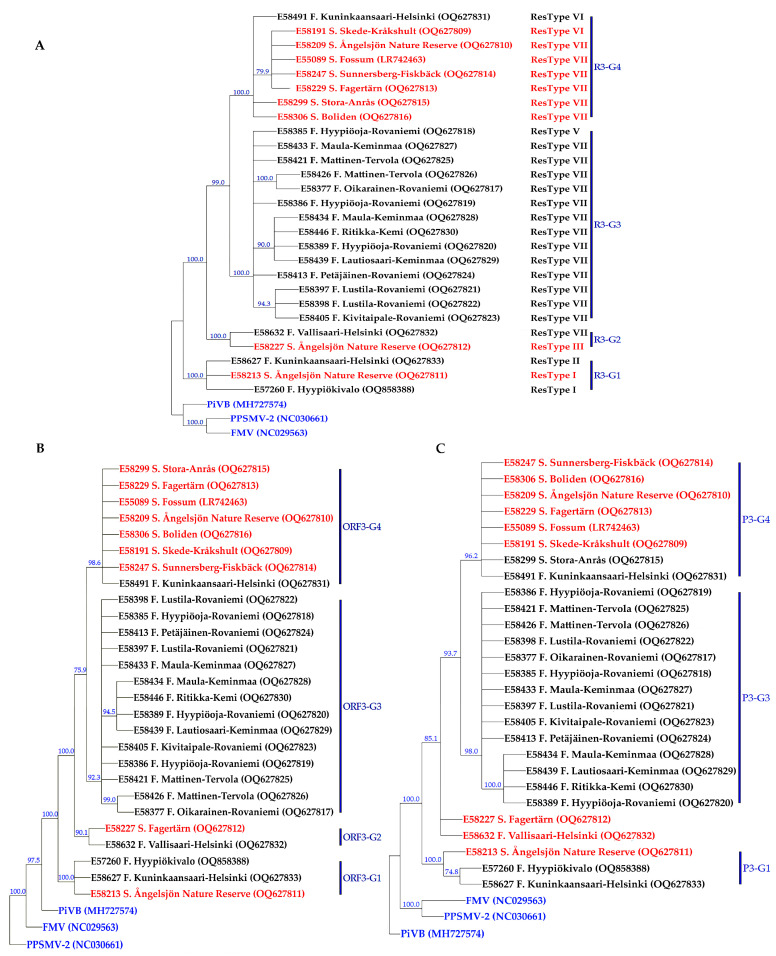
Consensus maximum parsimony trees constructed based on the nucleotide sequence of RNA3 (**A**), the open reading frame at nucleotide level 3 (**B**) as well as amino acid sequence of NP protein (**C**). Nodes with bootstrap support of less than 70% collapsed. The RNA3 sequences of pigeonpea sterility mosaic virus 2 (PPSMV–2) (*Emaravirus toordali*) (NC_030661), fig mosaic virus (FMV) (*Emaravirus fici*) (NC_029563), and pistacia virus B (PiVB) (*Emaravirus pistaciae*) (MH727574) were used as out-groups in the analyses. The NP protein sequences of PPSMV–2 (YP_009268864), FMV (YP_009237270), and PiVB (QAR18004) were used as out-groups in the amino acid-based tree of NP protein. The geographical origin and accession number of each isolate are presented next to the isolate name, as well as in Supplementary Table S3. Isolates from Sweden and Finland are shown in red and black colors, respectively, and the out-groups are in blue.

The substitution models of T92 + G and TN93 + G + I were selected as the best substitution models for AsMaV RNA3–4 and the partial RNA1, respectively, using the Find best DNA/Protein fit model test implemented in MEGA11 [49]. Similar tree topologies were obtained with both neighbor-joining and parsimony methods. Therefore, we only present and discuss the parsimony-generated trees. Based on the maximum parsimony tree generated from almost full-length 26 (18 Finnish and 8 Swedish) AsMaV RNA3 sequenced in this study and one previously reported isolate (LR742463) at the nucleotide level, AsMaV isolates clustered into four main groups. The first group, R3–G1, included E57260, E58627 (both from Finland), and E58213 (Sweden), and the second group, R3–G2, consisted of E58632 from Finland and E58227 from Sweden, group 3 (R3–G3) included the rest of the Swedish isolates (E58191, E58209, E58299, E58247, E58306, and E55089 with the E58491 isolate from Finland, and finally the rest of the Finnish isolates were placed in group 4 (R3–G4), i.e., E58377, E58385, E58386, E58397, E58398, E58405, E58413, E58421, E58433, E58434, and E58426. The identical grouping profile was documented when the tree was drawn based on the coding region of RNA3 (ORF3) at the nucleotide level. A similar tree, but with one difference was obtained when the phylogeny was based on the deduced amino acid sequences. In the latter tree, isolates E58227 (Sweden) and E58632 remained ungrouped. In general, isolates from Finnish and Swedish locations were separated based on their geographical origin with the exception of three isolates E58213 and E58227 from Sweden and E58491 from Finland based on the constructed phylogenetic trees obtained from RNA3. Interestingly, a robust correlation was observed between restrictotypes and phylogenetic groups, whereas no significant correlation was found between the geographical origin of the isolates and restrictotypes. Notably, restrictotypes I and II clustered together in the phylogenetic group R3–G1, restrictotypes III and IV formed a cohesive group in R3–G2, restrictotype V exhibited a close affinity with the Finnish isolates of restrictotype VII in R3–G3, and restrictotype VI demonstrated a close association with the Swedish isolates of restrictotype VII in R3–G4 (Figure 2 and Table 1).

### 3.3. Genetic Diversity and Phylogeny of AsMaV Isolates Based on RNA4

The length of RNA4 in sequenced isolates was 1557 nucleotides in size and there were no indel events. The length of 5′ UTR, ORF4, and 3′ UTR of RNA 4 were 86, 1086 (361 aa), and 385 nucleotides, respectively. The level of 95.1–99.9% and 95.8–99.4% identity on nucleotide level was estimated in pairwise comparisons of the isolates from Swedish and Finnish locations, respectively. Samples E58227 and E58209 from Sweden and the isolates E58421 and E58491 from Finland were the most divergent sequences of each geographical region. Like RNA3, the 3′ UTR was the most divergent region of the genomic RNA4 (Table 2).

The ORF4 in the sequenced isolates of AsMaV initiates with an AUG at position 87–90 and terminates with a UGA stop codon at position 1170–1172 (numbers based on the GenBank Acc. No. acc. no. LR742464). Interestingly, there were one or two UAA stop codons (in frame) in positions 1176–1181. This might denote that the size of MP plays an important role in the function of this protein. Alignment of the deduced amino acid sequences from published emaraviruses identified the following conserved amino acids or motifs: K_58_, Y_72_, R_88_I_89_XXXXXX(W/Y)_97_XP_98_, D_113_XR_115_, V_146_, D_168_I_169_X(K/Y)_171_(I/V)_172_, M_183_, W_197_XT_199_, (F/Y)_215_, E_224_, and (I/L)_326_. Similar motifs have been previously reported for PPSMV–1 and PPSMV–2, RLBV and RRV movement proteins and it has been suggested that some of these motifs showed a similarity to membrane-located protein and they might be involved in virus cell-to-cell movement [59,60].

**Figure 3:**
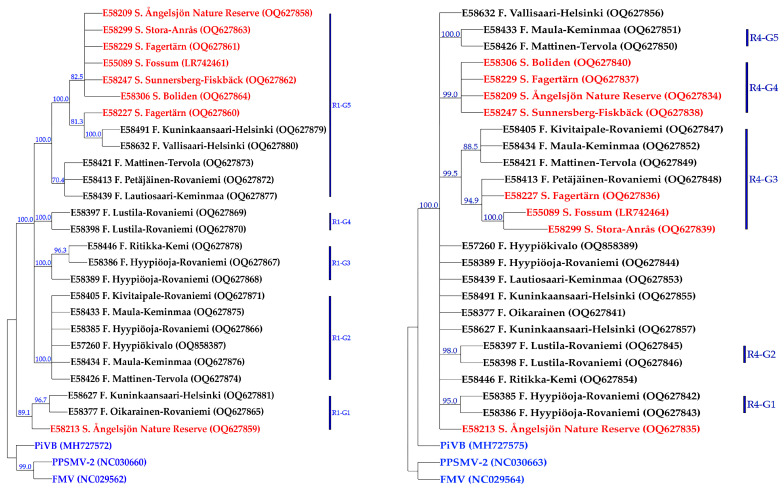
The consensus maximum parsimony trees constructed based on the nucleotide sequences of partial RNA1 (**left**) and RNA4 (**right**). Nodes with bootstrap support less than 70% were collapsed. Corresponding regions of RNA1 sequences of PPSMV–2, FMV, and PiVB with the accession numbers NC_030660, NC_029560, and MH727572, respectively, as well as those of full-length RNA4 with the accession numbers NC_030663, NC_029564, and MH727575, were used as out-groups in the analyses. The geographical origin and accession number of each isolate are presented next to the isolate name, as well as in Supplementary Table S3. Isolates from Sweden and Finland are shown in red and black colors, respectively. The out-groups are in blue.

In the CMP tree, constructed based on the AsMaV-RNA4, the 25 isolates sequenced in this study (18 from Finland and 7 from Sweden) and one previously reported isolate E55089 with 1000 bootstrap replicates at the nt level were placed in two main groups (R4–G1 and R4–G2). Isolate E58213 (Sweden) was placed in a separate clade close to the outgroups (FMV, PiVB, and PPSMV–2) (Figure 3). The R4–G1–A includes E58227, E58299, and E55089 from Sweden and an isolate from Finland, E58413. R4–G1–B includes three isolates from Finland, i.e., E58405, E58421, and E58434. The rest of the isolates from Sweden were placed as a clade in R4–G2 and the remaining Finnish isolates were placed in three subclades or remained ungrouped.

Most of the AsMaV isolates remained ungrouped in the CMP tree constructed based on the ORF4 at the amino acid level (data not shown). It might be that these sequences were not parsimoniously or phylogenetically informative. According to the CMD tree drawn based on the nucleotide sequence of ORF4, the Swedish isolates were grouped in two clades, ORF4–G2 and ORF4–G7, and isolate E58213 remained ungrouped. Five Finnish isolates were not placed in any group and isolate E58413 was placed between Swedish isolates in ORF4–G7. The rest of the Finnish isolates were grouped into five groups.

### 3.4. Genetic Diversity and Phylogeny of AsMaV Isolates Based on Partial RNA1

Based on the consensus maximum parsimony tree generated from 25 partial AsMaV RNA1 nt 20–1588 sequenced in this study (18 from Finland and 7 from Sweden) and one previously reported isolate (GenBank Acc. No. LR742461) at the nucleotide level, AsMaV isolates were placed in five main groups (Figure 3). Swedish isolates clustered in one clade (R1–G6) with the exception of E58213 which was grouped with the two Finnish isolates of E58632 and E58491 in the clade R1–G1. Interestingly, two Finnish isolates, E58632 and E58491, were clustered with the Swedish isolates in R1–G6. The isolates E58426, E58433, E58405, E58434, E57260, and E58385 in group R1–G2, isolates E58386, E58389, and E58446 in group R1–G3, isolates E58397 and E58398 in the group R1–G4, and the isolates E58413, E58421, and E58439 in the group R1–G5 (Figure 3). The phylogenetic tree based on the first 517 deduced aa sequences of AsMaV–ORF1 was not phylogenetically informative.

Among the generated phylogenetic trees for RAN3, RNA4, partial RNA1 at nt level, ORF3, ORF4, and partial ORF1 at both nucleotide and amino acid levels, phylogenetic trees constructed for AsMaV-RNA3 were in accordance with each other with one difference: isolates E58632 and E58227 remained ungrouped. There was no difference between phylogenetic trees generated based on the ORF3 or almost full length of the RNA3 at the nt levels. Therefore, it might be concluded that among investigated regions RNA3-based trees, regardless of full length (at nt level) or coding regions (at nt or aa level), produce almost the same results. AsMaV isolates were better discriminated in constructed phylogenetic trees based on the nt level of the AsMaV–RNA4 and partial RNA1 rather than aa-based trees, because approximately 57% (15 out of 26) and 30% (8 out of 26) of the isolates used for these analyses remained ungrouped in the mentioned phylogenetic trees, respectively. The phylogenetic trees based on the ORF4 in comparison to the almost full-length RNA4-based tree at nt levels had ungrouped isolates (six out of twenty-six). As mentioned before, both CMP trees constructed based on the partial nt sequence of RNA1 were identical. Therefore, using the nt sequences of almost full-length RNA3–4 and longer region(s) of RNA1 to generate phylogenetic trees was more informative than amino acid sequences of related ORFs (Figure 2 and Figure 3).

### 3.5. Complete Sequence of AsMaV-RNA1, RNA2 and RNA 5 of Distant Isolates

Based on the information provided, it appears that the isolates Kuninkaansaari, Finland), and E58632 (Vallisaari, Finland) were more divergent from compared isolates of AsMaV. This divergence is observed at both the nucleotide and amino acid levels in the pairwise alignment and their position in the constructed phylogenetic trees based on the RNA3, RNA4, and partial RNA1. To investigate this further, the complete RNA1, RNA2, and RNA5 of these isolates were amplified by RT–PCR, cloned, and sequenced.

The size of RNA1 was 7103 in E58627 and 7106 nucleotides in E58632, respectively. An indel event of three nucleotides was observed at positions 4033–4036, resulting in a shorter RdRP protein in E57627. However, the RdRP protein in these isolates still showed 98–99% similarity with the published isolate E55089 from Sweden at the amino acid level. E58632 and E55809 showed 97% identity at the nucleotide level, while E58627 showed 88% identity with E55809 and E58632. The ORF1 in the sequenced isolates of AsMaV initiates with an AUG at position 46–48 and terminates with an UGA stop codon at position 6955–6957 or 6952–6954 (numbers based on the GenBank Acc. No. LR742464). The RdRP was 2302 aa in isolate E58627 and 2303 in isolate 2303, respectively.

**Figure 4:**
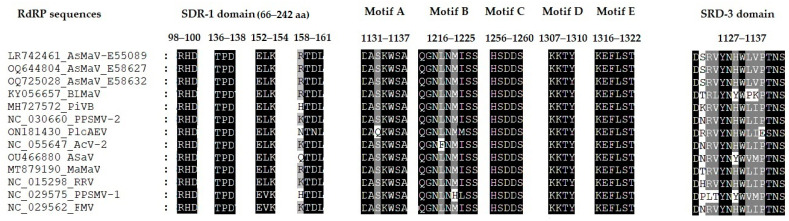
Motifs reported for RdRP of emaraviruses from phylogenetic clade A. SDR–1, SDR–2, and SDR–3 are the three main regions reported for RdRP in emaraviruses. SDR–1 domain contains the endonuclease motifs and might be involved in “cap–snatching”. SDR–2 contains the polymerase motifs designated motifs A–E, and SDR–3 is a Cap-binding domain.

Three different domains, N-terminus SRD–1, central region SRD–2, and C-terminus SRD–3 have been reported for RdRP in emaraviruses. The SRD–1 harbor endonuclease sequences like *Peribunyaviridae* polymerase and probably is involved in “cap-snatching”. It contains four motifs located between amino acids 66 and 242. The motifs RHD_98–100_ and TPD_136–138_ are universally conserved in different genera of *Peribunyaviridae* [61]. AsMaV RdRP sequences obtained in this study revealed that these motifs were in the same position and there were no changes in these motifs. But motifs EVK_152–154_ and KTDL_158–161_ vary across different genera [61]. It should be mentioned that the emaraviruses have been grouped based on their phylogenetic relations in four clades (A to D), in which AsMaV is placed in the clade A [6]. In clade A species of emaraviruses, the motif EVK_152–154_ has been reported for PPSMAV–1 and FMV and instead ELV_152–154_ was the dominant motif in most of the species including AsMaV (Figure 4). The KTDL_158–161_ motif was replaced by RKTDL_158–161_ in the RdRP of AsMaV isolates and could be R/H/N/Q/K_158_T_159_D/N_160_L_161_ in different species of Clade A species but its function is not yet determined.

The presence of six polymerase motifs (designated preA, A–E) characteristic of negative-strand RNA viruses [6,62] have been identified in the RdRP of AsMaV RNA1. This region was conserved in the sequenced isolates. 

A similar domain-like reported motif of DPLTYNYWVMPTN_1947–1959_ in the C-terminus of PPSMV–1 RdRP in the SRD–3 domain was identified as DSRVYNHWLVPTN in the AsMaV isolates sequenced in this study. Probably, this motif is a cap-binding site (CBS).

The RNA2 was 2287 nucleotides in length and ORF2 spanned on the nucleotide positions 58–1983 encodes for GPP (641 aa). There were no indel events. The RNA2 and GPP in isolates E58627 and E58632 shared 91% and 95% identity with E55089 at the nucleotide and amino acid levels, respectively. The two sequenced isolates shared 98% and 99% identity, respectively. It has been reported that the GPP of emaraviruses contains a N-terminal signal sequence, transmembrane domains, potential glycosylation sites, and a motif also found in the glycoprotein precursors of phleboviruses (family *Peribunyaviridae*) [37,63] which is processed by a protease into two glycoproteins, a smaller N-terminal one (Gn), and a larger C-terminal one (Gc) (Figure 1). The N-terminal region of the GPP precursor of AsMaV contains a signal peptide cleavage site, located at amino acid position 22 and 23 (AYT↓SIR) (SignalP–6.0 Prediction https://services.healthtech.dtu.dk/services/SignalP-6.0/, 12 April 2023). The similar cleavage site has been reported for PPSMV–1 at the same position but different sequence (AMA↓S) [63]. The glycoprotein contains four transmembrane helices (TMH) at amino acid positions 2–24, 106–128, 170–187, and 580–599 (TMHMM v.2.0 on https://services.healthtech.dtu.dk/services/TMHMM-2.0/, 12 April 2023). However, six glycosylation sites have been reported for emaraviruses, AsMaV had five out of six sites at the amino acid position 62, 201, 239, 328, and 438 which were predicted by NetNGlyc–1.0 (https://services.healthtech.dtu.dk/services/NetNGlyc-1.0/, 12 April 2023). Interestingly, the Mucin-type O-glycosylation site which is a widespread post-translational modification of proteins found in the entire animal kingdom, but also in higher plants, has been detected in divergent isolates of AsMaV identified in this study (E58627 and E58632) at the amino acid position 549. This was not detected in the previously published isolate of AsMaV. Interestingly, Mucin-type O-glycosylation sites have been detected in actinidia virus–2 (AcV–2), another species of clade A emaraviruses, in the positions 209, 254, and 264. It should be mentioned that Mucin-type O-glycosylation is an evolutionarily conserved protein modification reported for membrane-bound and -secreted proteins which are involved in protein secretion, stability, processing, and function [64]. More analyzing should be performed to understand the effects of these variations. 

The AsMaV RNA5 was 1324 nts in size in sequenced isolates of E58627 and E58632. In both isolates, the ORF5 initiated with a start codon of AUG at the positions 70–72 and terminated with a stop codon of UGA at the positions 811–813 encoding the P28 (247 aa) with unknown function. There was only one aa change in the divergent isolates in comparison to the reference isolate (E55809), S_240_ instead of N_240_. These sequences shared 98% and 99–100% identity with E55089 and each other, respectively, at nt and amino acid levels. It has been reported that the P28 of AsMaV shares a similar structure with ABC proteins [6].

### 3.6. Relation of Distant Isolates of AsMaV with Clade A Emaraviruses

AsMaV isolates shared a max. of 76%, 50%, 81%, and 80% identity in the pairwise alignment of RdRP, GPP, NP, and MP protein of clade A species of emaravirus in the RdRP, GPP, NP, and MP at the aa level being PiVB, PPSMV–1, PPSMV–2 the closest virus as confirmed with CMP trees. Therefore, the divergent isolates also belong to the AsMaV and did not belong to a new species.

**Figure 5:**
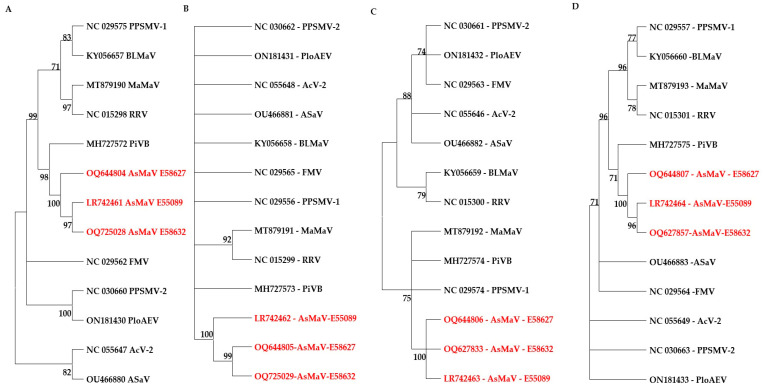
Constructed maximum parsimony trees based on the amino acid sequences of RdRP (**A**), GPP (**B**), NP (**C**), and MP (**D**) proteins. The evolutionary history was inferred using the Minimum Evolution method. The bootstrap consensus tree inferred from 1000 replicates is taken to represent the evolutionary history of the taxa analyzed. Branches corresponding to partitions reproduced in less than 70% of bootstrap replicates are collapsed. Trees were constructed by MEGA11 [49]. The AsMaV isolates are presented in red color.

In the phylogenetic tree based on the aa sequences of the encoded protein of AsMaV with corresponding proteins, the sequenced isolates of AsMaV were clustered in a distinct sub-cluster next to the PiVB. The GPP was not parsimoniously informative, since most of the species remained ungrouped. The RdRP-based tree had better discrimination of the species (Figure 5).

To study the phylogeny and genetic diversity of emaraviruses, researchers have utilized different regions of the emaravirus genome, each exhibiting varying degrees of variation among different emaravirus isolates. One particular region of interest is the NP due to its correspondence to the coat protein in non-enveloped viruses [29,30,31,32,33,34,35,36,37,38,39]. In certain emaraviruses, such as HPWMoV, the RNA3 or NP region has been identified as the most divergent genomic region, leading to the division of HPWMoV isolates into subgroups based on this region [28,39,65]. Similarly, for EMARaV, RNA3 exhibited more diversity than RNA4, and a phylogenetic tree based on RNA3 provided better discrimination among EMARaV isolates [38]. The NP has also been effectively used to assess genetic diversity and phylogenetic relationships among Serbian and Finnish isolates of RLBV, revealing a minimum of 93% identity [34]. In our study on AsMaV, our results indicate that the RNA3 or NP region is a promising candidate for investigating genetic diversity, followed by RdRP, which was found to be more phylogenetically informative than RNA4. For FMV, partial sequences of RNA1 to RNA4 were used to assess genetic diversity, revealing that RNA1 and RNA3 exhibited more variation than RNA4 [30]. Partial sequences of RNA1, RNA3, and RNA4 of MaMaV showed limited diversity, with RNA1 having a maximum of 2% variation and no diversity observed in the amplicons of RNA3 and RNA4 [32].

In contrast of our findings, it has been shown that the BLMaV-MP region displayed more variation than NP, possibly due to several detected recombination events in the coding region of BLMaV-MP [31]. Additionally, pairwise nucleotide comparisons of ORF1 to ORF6 of six JYMaV isolates revealed that RNA2 and RNA1 were the most divergent genomic regions when compared to ORF3 to ORF6 [36]. In the case of RRV, sequencing several full-length isolates demonstrated a conserved core genome, with RNA5 and RNA7 being the most divergent RNAs [35]. Notably, it is important to acknowledge that RRV-RNA5 is not equivalent to AsMaV-RNA5 due to inconsistencies in the nomenclature of genomic RNAs and proteins in emaraviruses [6].

The parsimony-based trees of the core genomic proteins of clade A species of emaraviruses indicated that RdRP is a better candidate for higher taxonomic levels (Figure 5). Partial RdRP at the nt level was also able to discriminate the AsMaV isolates. Similarly, most but not all of emaraviruses species could be grouped by amino acid sequences of NP. Therefore, it could be concluded that NP and RdRP are the best candidates for phylogenetic studies of emaraviruses for intra- and inter-species relationships, respectively. 

These examples highlight the diversity of genetic patterns across emaraviruses and underscore the uniqueness of each virus species in terms of their genomic characteristics and interactions with hosts. To gain a more comprehensive understanding of the genetic diversity and population structures of emaraviruses, increasing the number of available sequence data from more isolates, especially from distant geographical regions, would be beneficial. Such efforts can provide a clearer and more comprehensive picture of the evolutionary dynamics and relationships among emaraviruses.

### 3.7. Population Genetic Parameters

The estimated nucleotide diversity parameters for AsMaV populations in Finland and Sweden, including RNA3, RNA4, and partial RNA1 are represented in Table 3. As shown in Table 3, the AsMaV population from Finland had higher numbers of segregating sites compared to those from Sweden in the sequenced regions of the virus genome. This suggests that Finnish isolates are more genetically diverse than those in Sweden. The nucleotide diversity parameters show significant differences between RNA3 and RNA4 and partial RNA1 in both populations. In general, RNA4 ($\theta_{W}$ = 0.031979 and ${Pi}_{T}$ = 0.02738) has a lower level of nucleotide diversity than RNA3 ($\theta_{W}$ = 0.05876 and ${Pi}_{T}$ = 0.04994) and partial RNA1 ($\theta_{W}$ = 0.07509 and ${Pi}_{T}$ = 0.07777). These results also indicate that RNA3 and partial RNA1 populations are more diverse than RNA4 in both Finnish and Swedish populations.

For RNA3, Tajima’s D values were negative for both Sweden (−0.961799) and Finland (−0.961799), with the total also having a negative value (−0.579852). Fu and Li’s D* values were also negative. This suggests a recent population bottleneck or purifying selection, indicating an excess of rare isolates, which could be due to population subdivision or selection.

For RNA4, the population genetic parameters show relatively low levels of genetic diversity in both Swedish and Finnish populations. Again, the values of Theta ($\theta_{W}$) and ${Pi}_{T}$ are relatively low (0.02633 to 0.03198), indicating that there is not much genetic variation within these populations based on this region. The Tajima’s D value for Swedish isolates was positive, but it was not confirmed with negative values of Fu and Li’s D test. The Tajima’s D value and Fu and Li’s D test value for the Finnish population were both negative, indicating an excess of low-frequency isolates as a result of recent population expansion or purifying selection (Table 3).

For partial RNA1, the population genetic parameters show relatively high levels of genetic diversity in samples obtained from both countries, Sweden and Finland, with higher values of Theta ($\theta_{W}$ was 0.05930 to 0.07777) and ${Pi}_{T}$ (0.04447 to 0.08018) than in the other two genomic regions (Table 3). For partial RNA1, Tajima’s D values were negative for the Swedish population and positive for the Finnish population, with the total having a positive value. This suggests balancing selection or a recent population expansion. Fu and Li’s D* values were positive for Finnish isolates and negative for Swedish isolates and the total, indicating a possible selective sweep or population subdivision.

Overall, the population genetic parameters suggest that the genetic diversity in these populations was relatively low to moderate, with evidence of an excess of low-frequency isolates in some cases. These patterns could be due to recent population expansion or purifying selection. Additionally, natural selection may be acting to preserve protein function, as suggested by the low dn/ds ratio in some populations.

### 3.8. Population Differentiation Parameters

The results of population differentiation tests based on three different genomic regions of AsMaV (RNA3, RNA4, and Partial RNA1) among two different geographical groups, Swedish and Finnish isolates, are shown in Table 4. The null hypothesis was rejected for all differentiation tests, K_ST*_, Z*, and Snn, supported by *p*-values less than 0.05 (Table 4), denoting significant genetic differentiation and structure between the two groups in all the genomic regions. The F_ST_ values are moderate (ranging from 0.06 to 0.23), indicating that there is moderate genetic differentiation among populations, but some gene flow is still occurring. Overall, these results suggest that the virus populations of Sweden and Finland are genetically differentiated to some extent, but gene flow is still occurring. The moderate F_ST_ values suggest that there may be some environmental factors, such as geographic distance or barriers, influencing the genetic differentiation among the virus populations.

### 3.9. Recombination Analysis

The unstable position of a few isolates in phylogenetic trees raised the idea that there may be recombination events between the sequenced isolates. Searching for recombination events in the RNA4, RNA3, and partial RNA1 of AsMaV (nucleotides 20–1570) using RDP5 software revealed that there were no recombinant events in RNA4 and RNA3. However, a recombination event with a low probability has been detected by RDP (*E*-value 3.956 × 10^−3^), MaxChi (*E*-value 1.118 × 10^−2^), and Phylopro (*E*-value 2.545 × 10^−2^) in the isolate E58213 (Sweden) with the major parent of E58627 (Finland) and minor parent of E58299 (Sweden) in nts 581–631 and nts 647–915. It should be mentioned again that the E-value and the number of methods that supported this event were not strong. The isolate E58213 was placed in a different clade when the tree was based on the MP. The grouping profile of few isolates such as E58299, E58405, E58413, E58421, and E58434 were different. Since there were no recombination events reported for these isolates, two different scenarios would be possible: (i) there were insufficient data from AsMaV isolates especially from different geographical regions or (ii) the other mechanisms rather than recombination, for example, reassortment or genetic drift, were responsible for the evolution of these isolates.

Several recombination events have been reported in the HPWMoV-RNA3, especially in Australian isolates. It has been shown that recombination and reassortment plays an important role in the diversity of HPWMoV [39]. Therefore, it is concluded that in addition to mutation, the reassortment of gene segments and recombination within gene segments also contribute to the genetic diversity of emaraviruses [28]. Moreover, it has been suggested that certain FMV strains may have emerged through reassortments of genomic RNAs from different isolates [30].

## 4. Conclusions

Virus evolution involves changes in the genetic structure of a viral population over time, which begins with mutation, recombination, and reassortment. Then, host and vector factors, along with selection pressures, expose plant viruses to genetic drift, leading to the emergence of new viral strains or species with novel biological properties. Some viruses adapt to specific hosts or groups of hosts, while some can adapt to diverse hosts, vectors, and environments [23,24,25]. Therefore, studying the genetic diversity of viruses is important. Our understanding of the role of viruses in wild plant populations is improving, and we are beginning to understand how viruses affect the invasiveness of introduced plant species [66]. Since the wild trees, especially old ones, could survive even after being infected with viruses, we might consider these long interactions as symbiosis or tolerance of the wild trees to cope with these infections. However, viruses could also cause a decline in trees [67]. Most of the reported emaraviruses are attacking forest trees [6].

Our findings resolved that AsMaV–NP is the suitable candidate for genetic diversity studies of this virus since phylogenetic analysis based on full-length RNA3 or NP sequences resulted in similar profiles, and could discriminate the AsMaV isolates better than other genomic regions under investigation in this study. These results support the idea that the NP protein is a good representative of AsMaV genetic diversity studies. Although the RT–PCR–RFLP with AanI profiling did not correlate with the geographical origin of the isolates, it identified different haplotypes in Kuninkaansaari and Vallisaari islands, indicating the presence of multiple virus isolates in these islands as a hotspot for emerging new isolates. In addition, the number of the restriction site of AanI could be a landmark of emerging more divergent isolates of AsMaV since the isolates E58627, E58632, E58213, and E57260 were separated from other isolates of AsMaV in different phylogenetic trees. However, these were signals of recombination or reassortment in the phylogenetic trees based on different genomic regions of AsMaV isolates, thus, RDP analyses were not able to offer strong evidence. There is a need for sequence data from other geographical areas to obtain a better understanding of recombination events in AsMaV isolates. Recombination and reassortment would be also a mechanism involved in emerging new isolates, especially in the case of multipartite plant viruses [68] such as tospoviruses [69], and the cucumber mosaic virus (CMV) [19,20]. There are different reports on the frequency of reassortment in natural populations of plant viruses [19,20]. In the case of emaraviruses, it has been suggested that certain FMV strains may have emerged through reassortments of genomic RNAs from different isolates [30]. It has been shown that recombination and reassortment play an important role in the diversity of HPWMoV [39]. Therefore, it can be concluded that there is a need of sequence data from more AsMaV isolates, especially from more geographical regions. 

The genetic diversity parameters suggest that the Finnish population has higher genetic diversity compared to the Swedish population in all three analyzed genomic regions and these regions are under purifying selection. There is evidence of high selection pressure on AsMaV genome, but which was not consistent across different genomic regions. This pressure was almost equal on the N terminal region of RdRP (0.40) and MP (0.49), but it was at least five time less for NP (0.21). The nucleotide diversity of RNA3 and RNA4 of AsMaV were 0.059 and 0.32, respectively, which is higher than those reported for FMV (0.022 and 0.023) [30]. This range of nucleotide diversity was in a similar range for some other woody plant-invading viruses, such as CTV (0.038) and citrus leaf blotch virus (0.021) [30,70,71] but very low in comparison to grapevine fanleaf virus (0.15) [11,30,72,73]. The nucleotide diversity of blackberry leaf mottle-associated virus (BLMaV) was rather low when compared to FMV and European mountain ash ringspot-associated virus (EMARaV) [31]. Therefore, it could be concluded that the genomes of emaraviruses including AsMaV are relatively conserved.

The AsMaV isolates sequenced in this study were separated based on their geographical origin based on the phylogenetic trees. Deviations from the neutral equilibrium model for the three analyzed genes together with a combination of high haplotype diversity, overall low nucleotide diversity within Finnish and Swedish isolates, and negative values of Tajima’s and Fu and Li’s value indicate the existence of population expansions for AsMaV which is expected following genetic drift, which accompanies bottleneck transmission [74]. Evolutionary bottlenecks/founder effects may arise as a result of plant- or vector-associated effects [75]. Root suckering will generally occur in the aspen stands as they deteriorate or as they are disturbed by fire or other events. In addition, some species of gall mites have been identified as a natural vector for a few emaraviruses and for most emaraviruses such a natural vector has not been identified so far [6]. Occurrence of population expansions following a bottleneck event for other multi-segmented plant RNA virus have been reported [76], and suggest that the inferred differentiation between viral populations is because of demographically related events.

The Finnish and Swedish AsMaV isolates sequenced in this study displayed geographical structuring that may have arisen by founder effects. The purifying selection acts on low to moderate genetic diversity of AsMaV isolates to preserve function of the protein. Since emaraviruses have a narrow host range and, in most of reported emaraviruses, they have been reported only from one natural host, it might reduce some factors affecting the shape of the population in comparison with viruses with a wider host range. Only a few reports are available for natural vectors of emaraviruses. The reported mites have also a narrow host range which could subsequently cause the lower genetic diversity of the emaraviruses. In this context, the most diversity in AsMaV isolates was found in Kuninkaansaari and Vallisaari islands. There is a need for investigation of the mite species found on *Populous* spp. and their potential in transmissions of AsMaV. Distant isolates of AsMaV showed some changes in aa sequences in comparison to other isolates and bioinformatic data predicted a post-translational Mucin-type O-glycosylation site in GPP which could happen because of interactions with the natural vector. Further studies are needed to investigate whether the mutations identified in the sequence variants of AsMaV RNA1, RNA3, and RNA4 affect the virus replication cycle, the severity of symptoms expressed by virus, may contribute to the decline in trees, or have an impact on the interaction with the assumed mite vectors of the virus. The functional characterization of the AsMaV-host–vector relationships will contribute to our understanding of the ecological position of the virus, and are a prerequisite to handle and prevent the damages including the decline that may be caused by the virus, especially when considering additional stress factors such as the predisposition and impacts of climate change.

## Figures and Tables

**Table 1:** Position and frequency of AanI restriction site on AsMaV–RNA3 of 34, 47, and 81 collected samples from Finland (F), Sweden (S), and in total (T), respectively. The position of the nucleotides is based on AsMaV–RNA3 sequence of isolate E55089 (GenBank accession number LR742463). The black line represents the genomic RNA and orange rectangle shows the position of the ORF3, and the vertical red lines are denoting the position of AanI restriction site on the RNA3 (nucleotides: 627, 1069, 1142, 1406, 1474).

Restrictotype	Position of AanI Site on cRNA3	No. Cut ^1^	No. Fr. ^2^	Size of Fragments (bp)	Frequency of Restrictotypes (%) ^3^		
					S	F	T
Type I	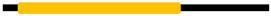	0	1	1587 to 1589	1.2	6.1	7.3
Type II	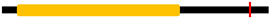	1	2	1474, and 115	0.0	1.2	1.2
Type III	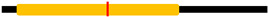	1	2	962, and 627	2.4	3.7	6.1
Type IV	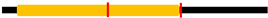	2	3	627, 521, and 442	0.0	4.9	4.9
Type V	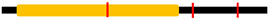	3	4	627, 515, 266, and 182	1.2	2.4	3.7
Type VI	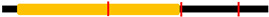	3	4	627, 442, 338, and 182	3.7	1.2	4.9
Type VII	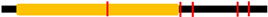	4	5	627, 442, 264, 182, and 73	32.9	39.0	72.0

^1^ number of DNA restriction enzyme sites; ^2^ number of fragments recorded on electrophoresis after digestion of the RT–PCR with AanI restriction enzyme; ^3^ the percentages of each restrictotype were calculated based on the total number of collected isolates in this study, the list of which, along with their corresponding restrictotype groups, is presented in Supplementary Table S1 and the geographical distribution of restrictotypes is presented in the Supplementary Figure S1.

**Table 2:** The identities of the RNA3, RNA4 and partial RNA1 at nucleotide (nt) and amino acid (aa) levels among the 18 Finnish and seven to eight Swedish isolates sequenced in this study.

Origin of Isolates		Genomic Region							
		RNA3			RNA4			Partial RNA1	
		5′ UTR	ORF3	3′ UTR	5′ UTR	ORF4	3′ UTR	5′ UTR	ORF1
Finland	nt	95–100	88.9–100	80.8–98.8	97.6–100	95.9–99.9	82.4–100	96.5–100	85.2–99.7
	aa		**88.4–100**			**96.9–100**			**96.6–100**
Sweden	nt	94–100	89.4–99.6	80–98.8	100	95.9–99.9	80–98.8	97.6–100	87.4–98.7
	aa		**91.8–100**			**97.5–100**			**97.1–99.7**

Values for amino acid level are shown in bold.

**Table 3:** Results from population parameters estimated based on demographic trends.

Genomic Regions	Geographical Group	m	S	$\theta_{w}$	${Pi}_{T}$	${Pi}_{s}$	${Pi}_{a}$	${Pi}_{a}/{Pi}_{s}$	Tajima’s D	Fu and Li’s D
**RNA3**										
	Sweden	9	275	0.06347 ± (0.02651)	0.04735 ± (0.01764)	0.11984	0.02115	0.176	−1.322703	−1.56810
	Finland	18	298	0.05435 ± (0.01881)	0.04207 ± (0.01138)	0.11180	0.02432	0.217	−0.961799	−0.47563
	Total	27	361	0.05876 ± (0.01844)	0.04994 ± (0.00834)	0.12843	0.02695	0.210	−0.579852	−0.60644
**RNA4**										
	Sweden	8	119	0.02948 ± (0.01286)	0.03048 ± (0.00391)	0.09485	0.00542	0.057	0.186391	−0.14740
	Finland	18	141	0.02633 ± (0.00929)	0.02479 ± (0.00237)	0.08977	0.00418	0.047	−0.246562	−0.29650
	Total	26	190	0.03198 ± (0.01032)	0.02738 ± (0.00187)	0.09406	0.00460	0.049	−0.568813	−0.66359
**Partial RNA1**										
	Sweden	8	242	0.05930 ± (0.02564)	0.04447 ± (0.01850)	0.19665	0.00934	0.048	−1.362813	−1.50039
	Finland	18	417	0.07702 ± (0.02673)	0.08018 ± (0.00753)	0.40730	0.01603	0.039	0.174721	0.10686
	Total	26	451	0.07509 ± (0.02390)	0.07777 ± (0.00663)	0.39134	0.01554	0.040	0.153180	−0.04648

The abbreviations used are as follows: m, number of sites; S, number of segregating sites; $\theta_{W}$ = Waterson’s Theta; ${Pi}_{T}$ = total nucleotide diversity; ${Pi}_{s}$ = the number of synonymous substitutions per synonymous site; ${Pi}_{a}$ = the number of nonsynonymous substitutions per nonsynonymous site.

**Table 4:** Geneflow and genetic differentiation parameters.

Genomic Region	Populations	K_ST*_	*p*-Value	Z*	*p*-Value	Snn	*p*-Value	Hs	*p*-Value	F_ST_
**RNA3**	S. vs. F	0.07092	0.0000 ***	4.51006	0.0000 ***	0.85185	0.0010 **	0.99545	0.5330 ^ns^	0.22767
**RNA4**	S. vs. F	0.02288	0.0080 **	4.63422	0.0050 **	0.88462	0.0030 **	1.00000	1.0000 ^ns^	0.06408
**RNA1**	S. vs. F	0.03890	0.0000 ***	4.44027	0.0000 ***	0.92308	0.0010 *	1.00000	1.0000 ^ns^	0.23698

^ns^ not significant, * 0.01 < *p*-value < 0.05; **, 0.001 < *p*-value < 0.01; ***, *p*-value < 0.001.

## Data Availability

The sequence data have been submitted to GenBank.

## Supplementary Materials

The following supporting information can be downloaded at: https://www.mdpi.com/article/10.3390/v15081678/s1, Table S1: list of collected samples; Table S2: characteristics of the oligonucleotides used in this study; Table S3: (a) the GenBank accession numbers of the isolates sequenced in this study; (b) the GenBank Accession numbers for the complete genome. Figure S1: distribution of AanI restrictotypes of AsMaV-RNA3 on a geographical map.
