# Supplementary material for: Molecular Population Genetics of Aspen Mosaic-Associated Virus in Finland and Sweden
Source: Viruses. 2023 Aug 1;15(8):1678. doi: 10.3390/v15081678 (PMC10460043; doi:10.3390/v15081678)
Supplement: Supplementary file 1 [file viruses-15-01678-s001.zip › Supplementary Figure S1.pptx]

## Slide 1
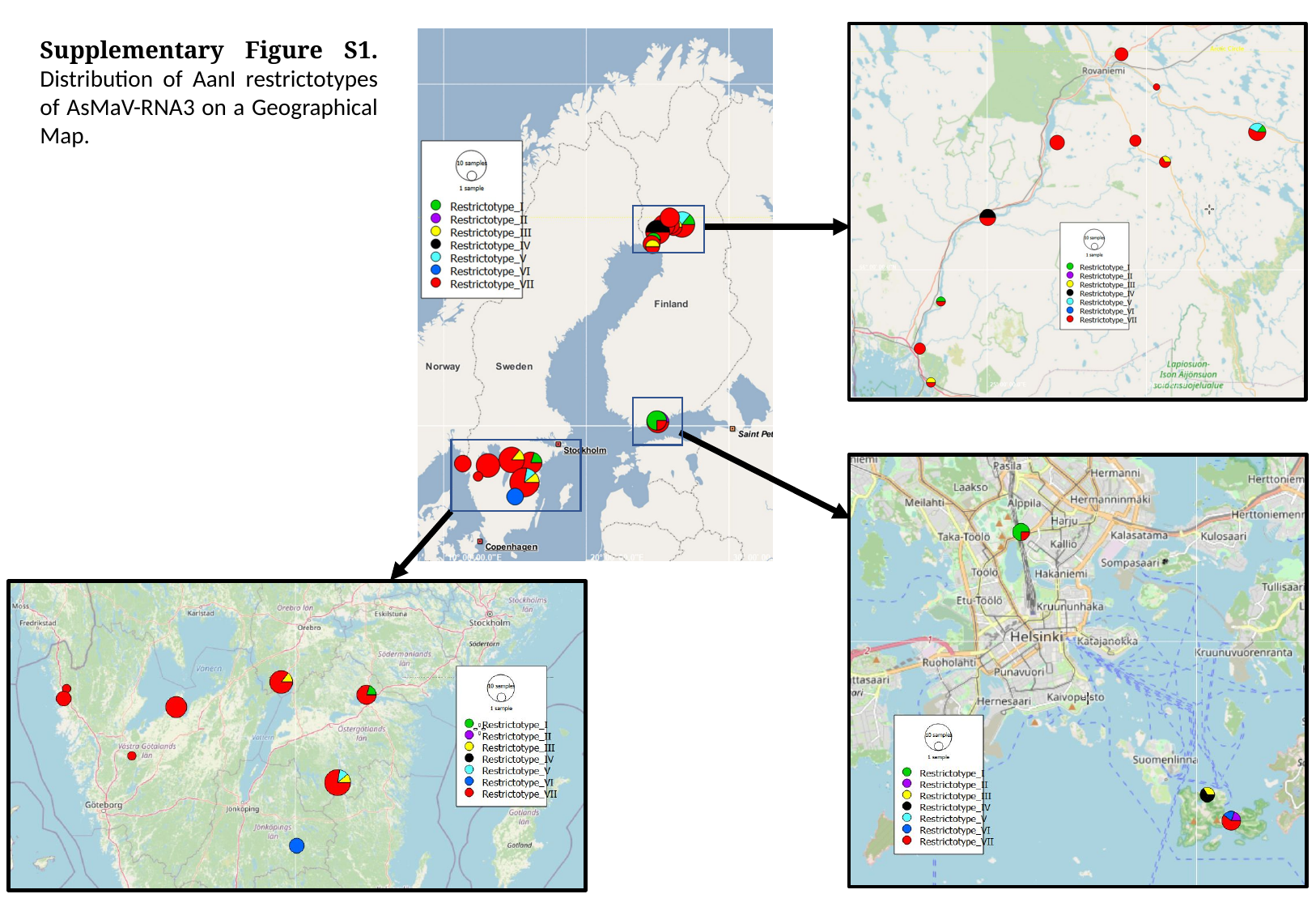

Supplementary Figure S1. Distribution of AanI restrictotypes of AsMaV-RNA3 on a Geographical Map.
