# Supplementary material for: Molecular Population Genetics of Aspen Mosaic-Associated Virus in Finland and Sweden
Source: Viruses. 2023 Aug 1;15(8):1678. doi: 10.3390/v15081678 (PMC10460043; doi:10.3390/v15081678)
Supplement: Supplementary file 1 [file viruses-15-01678-s001.zip › Supplementary Table S2. Characteristics of the oligonucleotides.pdf]

Supplementary Table S2. Characteristics of the oligonucleotides used in this study. The RNA1 to RNA5 sequences of AsMaV isolates E55089 (GenBank accession No. LR74246, LR742462–65) were used for hybridization regions of the primers to the genomic cRNAs.

Primer name	Sequence 5' → 3'	RNA	Position	Reference
**Primers used for detection**				
motif-A-sense	GATGCATCDAAATGGTCWGC	1	3436–3455	[47]
motif-C-antisense	ATCATCWGARTGHACCAT	1	3822–3805	[47]
AsMaV1-F	GATGATTATCTCCTAACTAGG	1	5583–5563	[5]
AsMaV1-R	GTGATGGCCTGTTAAGAATTC	1	5790–5770	[5]
AsMaV2-F	CAATATCAATCTGTAAGGGTG	2	272–292	[5]
AsMaV2-R	CATTGTCATCAGCCTTGACG	2	634–615	[5]
AsMaV3-F	GAGAAGCCTCAGATTTACTGA	3	202–222	[5]
AsMaV3-R	CACATGCTTTGTTGAATGAGAC	3	518–397	[5]
AsMaV4-F	GTTCCCAAGTACGAGTGGAA	4	125–144	[5]
AsMaV4-R	AGTGTTGCCATCTCCTGGA	4	412–394	[5]
AsMaV5-F	ATGGAGCTAAAAGCGTTCGAA	5	70–90	[5]
AsMaV5-R	GAACAACTTGCTTCTTGGTCT	5	714–694	[5]
**Primers used for cloning**				
PDAP213	GGCGACCCGCTCCGGTACCCTAGTAG TGAACTCC	1–5	13 conserved terminal nucleotides (underlined)	[48]
AsMaV-R1AandE-1-s	AGTAGTGAACTCCCTTTAATAC	1	1–22	This study
AsMaV-R1A-1627-as	CACTCACCAGATCATTTGC	1	1645–1627	This study
AsMaV-R1B-1541-s	CCATGGCTGATTCAGAGAACG	1	1541–1561	This study
AsMaV-R1B-3107-as	TGGTATTCATCAACGAGTC	1	3107–3125	This study
AsMaV-R1C-2808-s	TGACAATATRGACATACATGCA	1	2808–2829	This study
AsMaV-R1C-4692-as	CTTYTTYACACACCATTCAGGA	1	4692–4713	This study
AsMaV-R1D-4435-s	ATACAGGATTATCTAGATCTC	1	4435–4455	This study
AsMaV-R1D-6011-as	TTTTGTTGGCTAAATGTCC	1	6011–6029	This study
AsMaV-R1E-5968-s	TCTCTATCRTATTACATGTG	1	5968–5987	This study
AsMaV-RNA3-18-s	CCAGTAGTGAACTCCCATTA	3	1–18	This study
AsMaV-RNA3-end-as	AGTAGTGAACTCCCATTATAC	3	1587–1569	This study
AsMaV-RNA4-12-s	CCTTACAACAAGAATCAACTG	4	12–32	This study
AsMaV-RNA4-end-as	AGATCTAGTAGTGAACTCCTTAC	4	1558–1541	This study
**Primers used for sequencing**				
AsMaV-R2-897-s	CAGAAAGGCTCATGATGGGT	2	897–916	This study
AsMaV-R3-753-s	TGAGGCTAGAGTACAGGAAG	3	753–772	This study
AsMaV-R3-962-as	GACCTCTATAGCATCATCATT	3	962–982	This study
AsMaV-R4-845-as	GAGAGAAGTTGTTCAGCTGC	4	845–864	This study
AsMaV-R4-745-s	GACTCCCTGCTGAAATTGAGC	4	745–765	This study
