# Supplementary material for: Molecular Population Genetics of Aspen Mosaic-Associated Virus in Finland and Sweden
Source: Viruses. 2023 Aug 1;15(8):1678. doi: 10.3390/v15081678 (PMC10460043; doi:10.3390/v15081678)
Supplement: Supplementary file 1 [file viruses-15-01678-s001.zip › Supplemntary Table S3 GenBank Acss.pdf]

Supplementary Table S3. (a): the GenBank accession numbers of the isolates sequenced in this study.

Isolate	Country	Isolation source	GenBank Accession		
			RNA3	RNA4	partial RNA1
E58191	Sweden	Skede-Kråkshult	OQ627809	------	------
E58209	Sweden	Ångelsjön Nature Reserve	OQ627810	OQ627834	OQ627858
E58213	Sweden	Ångelsjön Nature Reserve	OQ627811	OQ627835	OQ627859
E58227	Sweden	Fagertärn-road	OQ627812	OQ627836	OQ627860
E58229	Sweden	Fagertärn-road	OQ627813	OQ627837	OQ627861
E58247	Sweden	Sunnersberg-Fiskbäck	OQ627814	OQ627838	OQ627862
E58299	Sweden	Stora-Anrås	OQ627815	OQ627839	OQ627863
E58306	Sweden	Boliden	OQ627816	OQ627840	OQ627864
E58377	Finland	Oikarainen-Rovaniemi	OQ627817	OQ627841	OQ627865
E58385	Finland	Hyypiöoja-Rovaniemi	OQ627818	OQ627842	OQ627866
E58386	Finland	Hyypiöoja-Rovaniemi	OQ627819	OQ627843	OQ627867
E58389	Finland	Hyypiöoja-Rovaniemi	OQ627820	OQ627844	OQ627868
E58397	Finland	Lustila-Rovaniemi	OQ627821	OQ627845	OQ627869
E58398	Finland	Lustila-Rovaniemi	OQ627822	OQ627846	OQ627870
E58405	Finland	Kivitaipale-Rovaniemi	OQ627823	OQ627847	OQ627871
E58413	Finland	Petäjäinen-Rovaniemi	OQ627824	OQ627848	OQ627872
E58421	Finland	Mattinen-Tervola	OQ627825	OQ627849	OQ627873
E58426	Finland	Mattinen-Tervola	OQ627826	OQ627850	OQ627874
E58433	Finland	Maula-Keminmaa	OQ627827	OQ627851	OQ627875
E58434	Finland	Maula-Keminmaa	OQ627828	OQ627852	OQ627876
E58439	Finland	Lautiosaari-Keminmaa	OQ627829	OQ627853	OQ627877
E58446	Finland	Ritikka-Kemi	OQ627830	OQ627854	OQ627878
E58491	Finland	Kuninkaansaari-Helsinki	OQ627831	OQ627855	OQ627879
E58627	Finland	Kuninkaansaari-Helsinki	OQ627832	OQ627856	OQ627880
E58632	Finland	Vallisaari-Helsinki	OQ627833	OQ627857	OQ627881
E57260	Finland	Hyypiökivalo	OQ858388	OQ858389	OQ858387

(b): the GenBank Accession numbers for the complete genome.

Isolate	Country	Isolation source	genomic region	Acc. No.
E58627	Finland	Kuninkaansaari-Helsinki	RNA1	OQ644804
E58627	Finland	Kuninkaansaari-Helsinki	RNA2	OQ644805
E58627	Finland	Kuninkaansaari-Helsinki	RNA3	OQ644806
E58627	Finland	Kuninkaansaari-Helsinki	RNA4	OQ644807
E58627	Finland	Kuninkaansaari-Helsinki	RNA5	OQ644808
E58632	Finland	Vallisaari-Helsinki	RNA1	OQ725028
E58632	Finland	Vallisaari-Helsinki	RNA2	OQ725029
E58632	Finland	Vallisaari-Helsinki	RNA3	OQ627833
E58632	Finland	Vallisaari-Helsinki	RNA4	OQ627857
E58632	Finland	Vallisaari-Helsinki	RNA5	OQ725030
